# Circ-PKD2 promotes Atg13-mediated autophagy by inhibiting miR-646 to increase the sensitivity of cisplatin in oral squamous cell carcinomas

**DOI:** 10.1038/s41419-021-04497-8

**Published:** 2022-02-26

**Authors:** Ling Gao, Qian Zhang, Shaoming Li, Jingjing Zheng, Wenhao Ren, Keqian Zhi

**Affiliations:** 1 Department of Oral and Maxillofacial Surgery, The Affiliated Hospital of Qingdao University, Qingdao, Shandong China; 2 Key Lab of Oral Clinical Medicine, The Affiliated Hospital of Qingdao University, Qingdao, Shandong China; 3 School of Stomatology of Qingdao University, Qingdao, Shandong China; 4 Department of Stomatology, The Affiliated Hospital of Qingdao University, Qingdao, Shandong China

**Keywords:** Diagnostic markers, Oral cancer

## Abstract

Autophagy is an evolutionally conserved catabolic process that degrades cells to maintain homeostasis. Cisplatin-activated autophagy promotes the expression of circ-PKD2, which plays a role as a tumor suppressor gene in the proliferation, migration, and invasion in oral squamous cell carcinoma (OSCC). However, the role of circ-PKD2 in regulating the sensitivity of OSCC patients to cisplatin remains to be elucidated. Overexpression of circ-PKD2 increased the formation of autophagosomes in OSCC cells and activation of proteins, such as LC3 II/I. Its activation effect on autophagy was, however, alleviated by 3-MA. Bioinformatics analyses and double luciferases reporter assays conducted in this study confirmed the existence of targeted relationships between circ-PKD2 and miR-646 and miR-646 and Atg13. Functional experiments further revealed that miR-646 reversed the autophagy and apoptosis effects of circ-PKD2 in OSCC cells treated with cisplatin. In addition, circ-PKD2 promoted the expression of ATG13 by adsorption of miR-646. Its interference with Atg13 alleviated the activation effects of circ-PKD2 on autophagy and apoptosis of miR-646. Notably, the in vivo animal experiments also confirmed that circ-PKD2 inhibited tumor proliferation and activated autophagy in OSCC cells. This study provides a theoretical basis for using circ-PKD2 as a target to regulate the sensitivity of OSCC patients to cisplatin, thus increasing its chemotherapeutic effects.

## Introduction

Oral squamous cell carcinoma (OSCC) is a common head and neck malignant tumor. It has a high prevalence and incidence, with 300,000 new cases and 145,000 deaths attributed to OSCC reported globally every year [1]. Cisplatin is the first-line chemotherapy drug for OSCC patients. However, 30 percent of patients are initially insensitive to cisplatin. Moreover, patients who are initially sensitive to the drug develop varying degrees of drug resistance after three or four rounds of chemotherapy [2, 3]. It is thus essential to develop a novel strategy to enhance the sensitivity of OSCC to cisplatin.

Autophagy is a conservative cell degradation process that can maintain normal physiological activities and homeostasis in cells, causing them to rapidly adapt to pressure or hostile environment [4]. Notably, activation of autophagy through standard chemotherapy contributes to chemotherapy resistance in specific cancer environments. For instance, autophagy inhibition combined with chemotherapy significantly increased the death rate of tumor cells, promoting the pro-survival role of autophagy in promoting tumor resistance to chemotherapy [5]. Recent studies postulate that autophagy is elevated in cisplatin-resistant osteosarcoma cells. However, pharmacological inhibition of autophagy by 3-MA significantly increases their sensitivity to cisplatin [6]. Currently, the role and mechanism of autophagy in OSCC sensitivity to chemotherapy remains to be fully elucidated.

CircRNA is a novel covalently closed non-coding RNA that regulates gene expression in eukaryotes [7]. There is growing evidence that circRNAs are associated with various diseases, such as neuro dystrophy [8], cardiovascular disease [9–11], and cancer [12, 13], despite being lowly expressed. Recently, circRNAs have been reported to be abundant and stable in plasma [14], saliva [15, 16], and even serum exosomes [17], suggesting that they are potential readable biomarkers. Previous studies demonstrated that circ-PKD2 was significantly down-regulated in OSCC tissue samples. Notably, overexpression of circ-PKD2 significantly inhibits the proliferation, migration, and invasion of OSCC in vitro [18]. However, the involvement of circ-PKD2 in regulating the sensitivity of OSCC to cisplatin remains unknown.

This study revealed that circ-PKD2 promoted the sensitivity of OSCC to cisplatin both in vitro and in vivo. Functional experiments further demonstrated that the expression of circ-PKD2 and autophagy-related proteins increased in OSCC cells treated with cisplatin. We thus hypothesize that circ-PKD2 potentially regulates the sensitivity of OSCC cells to cisplatin by upregulating autophagy. In the same line, bioinformatics analyses revealed that circ-PKD2 targets miR-646, which inhibited ATG13, thus increasing the expression of ATG13. In this study, circ-PKD2 targeted miR-646, thus up-regulated autophagy which promoted the sensitivity of OSCC cells to cisplatin, This study provides a theoretical basis for circ-PKD2 as a target to regulate the sensitivity of OSCC patients to cisplatin, thus increasing its chemotherapeutic effects.

## Materials and methods

### Cell culture and transfection

The human OSCC cell lines SCC-15 and CAL-27 were purchased from the Cell Bank of Type Culture Collection of the Chinese Academy of Sciences (Shanghai, China). They were cultured in Dulbecco’s Modified Eagle’s medium (HyClone, USA) supplemented with 10% fetal bovine serum (FBS) and 1% penicillin/streptomycin at 37 °C in a humidified atmosphere containing 5% CO_2_. An overexpression plasmid of circ‐PKD2 containing the green fluorescent protein gene was acquired from HANBIO (Shanghai, China). The si‐circ‐PKD2, si-miR-646, miR‐646 mimics and si-Atg13 were designed and produced by GenePharma (Shanghai, China). The sequences were as follows. miR-646 mimics: 5′-AAGCAGCUGCCUCUGAGGCCUCAGAGGCAGCUGCUUUU-3′; miR-646 inhibitor: 5′-GCCUCAGAGGCAGCUGCUU-3′; si‐circ‐PKD2: 5′-GTGTATTGACCTACGGCATGA-3′; si-Atg13: 5′-AAGUCCCUUCUUGCUAUAACUAGTTCUAGUUAUAGCAAGAAGGGACUUTT-3′. si‐circ‐PKD2, si-miR-646, miR‐646 mimics, and si-Atg13 were transfected into SCC-15 and CAL-27 cell lines using Lipofectamine 3000 (Thermo Fisher Scientific) transfection reagent according to the manufacturer’s protocol.

### RNA extraction and qRT‐PCR

Total RNA was extracted from the cells using TRIzol reagent (Takara Bio Inc, Japan). The quantitative real-time polymerase chain reaction (qRT-PCR) was performed using a Prime Script RT reagent kit (TaKaRa Bio Inc) and SYBR Premix Ex Taq II (TaKaRa Bio Inc) in the Bio-Rad CFX96 PCR machine according to the manufacturer’s instruction. GADPH or U6 were used for normalizing the RNA expression levels in qRT‐PCR, and the relative expression was calculated using the 2^−ΔΔCt^ method. All the qRT‐PCR reactions were run in triplicates. Primers used in this study are listed in Additional file 1: Table S1.

### Western blotting analysis

Proteins were extracted with RIPA lysis buffer (Beyotime Biotechnology, Shanghai, China) according to the manufacturer’s instructions. The concentrations of the extracted proteins were determined using the BCA protein assay kit (Pierce, USA). For each protein sample, an equal amount was subjected to 8–12% sodium dodecyl sulfate-polyacrylamide gel electrophoresis gel and then transferred to polyvinylidene fluoride (PVDF) membranes (Merck Millipore, Billerica, MA, USA). The membranes were blocked in 5% non-fat milk for 2 h, after which they were incubated with primary antibodies at 4 °C overnight. Then, the membranes were incubated with horseradish peroxidase-conjugated antibodies for 1 h at room temperature. Finally, the target protein bands were detected using the ChemiDoc Touch Imaging System (BioRad), and the band’s intensity was analyzed using the ImageJ program (National Institutes of Health, Bethesda, MD). The antibodies for the western blotting were Atg13 (#13468, CST), Cleaved Caspase-8 (#9496, CST), Caspase-8 (#9746, CST), Cleaved caspase-3 (#9661, CST), P62/SQSTM1 (18420-1-AP, proteintech), LC3 (14600-1-AP, proteintech) and GAPDH (#5174, CST).

### Transmission electron microscopy (TEM)

The SCC-15 and CAL-27 cell colonies were fixed in 2.5% glutaraldehyde phosphate-buffered saline and post-fixed in 1% osmium tetroxide. The cells were then dehydrated along a gradient of increasing ethanol and propylene oxide concentrations and embedded. They were then cut into 50 nm sections and stained with 3% uranyl acetate and lead citrate, which were observed under the JEM-1010 transmission electron microscope (JEOL, Tokyo, Japan). The number of cells per section was counted.

### Luciferase reporter assay

A total of 5 × 10^4^ cells were inoculated into 24-well plates and co-transfected with wild-type (WT) or mutated type (MUT) circ-PKD2 or Atg13 3′UTR report plasmid and miR-646 mimics or negative control using Lipofectamine 3000 following the manufacturer’s instruction. the relative luciferase activity was measured 48 h after transfection using a Dual Luciferase Assay Kit (Promega) according to the manufacturer’s protocol.

### CCK-8 and colony formation assays

The cells were seeded into 96-well cell culture plates (4000 cells per well) containing the culture medium with different concentrations of cisplatin (CDDP) and incubated for 24 h. Afterward, 10 μl CCK8 solution (Dojindo Laboratories, Tokyo, Japan) was added to each well was and incubated for 2 h at 37 °C in the cell incubator. The absorbance in each well was measured using a spectrophotometric plate reader (Molecular Devices, Sunnyvale, CA, USA) at OD450. Each group had five replicates, and the CCK-8 assay was repeated thrice. Then, the IC50 values for cisplatin were calculated. For colony formation assays, 1000 cells were plated in 6-well plates in 2 ml of culture medium and incubated for 12 h, after which cisplatin was added. The plates were maintained for 1–2 weeks; then, the colonies were washed.

### Immunohistochemical (IHC) analysis

The tissue sections were dewaxed with xylene and treated with gradient ethanol, after which they were treated with a citric acid antigen repair solution. The goat serum was then added and maintained for 10 min, followed by drops of working antibody solution (1:100), and incubated overnight at 4 °C. DAB and hematoxylin staining were performed on the second day. Finally, the dehydrated slices were sealed, observed under the Olympus BX53 microscope (Olympus, Tokyo, Japan), and images were generated using the same microscope.

### Apoptosis analysis

A total of 1 × 10^6^ cells were digested by trypsin, then washed in PBS, and resuspended in 1 ml binding Buffer. Then 100 μl cell suspension was added to the EP tube, and 5 μl of Annexin V-FITC and PI (Solarbio, China) were added and incubated in the dark for 10 min room temperature. The volume was adjusted to 500 μl with PBS, and the cell apoptosis was analyzed using a flow cytometer (Beckman Coulter, Palo Alto, CA, USA).

### Immunofluorescence (IF) analysis

SCC-15 and CAL-27 cells (1 × 10^4^) were seeded into a coated glass-bottom dish and maintained for 24 h after which DMEM was removed. The cells were then washed in PBS for 2 min, then fixation with 4% paraformaldehyde for 10 min at room temperature, and the dish was washed in three changes of PBS. Next, PBS containing 0.2% Triton-X-100 was added for 30 min at room temperature to permeate the cell membrane. The cells were washed in three changes of PBS solution, then blocked in 5% bovine serum albumin for 30 min at room temperature. Next, the cells were incubated with primary antibodies (1:200) overnight at 4 °C under gentle shaking. The cells were washed with PBS solution and incubated with FITC-conjugated secondary antibodies in the dark for 1 h at room temperature. At last, the cells were mounted with an antifading mounting medium with DAPI and photographed using a fluorescence microscope (DMI3000B, Leica microsystem, Germany).

### In vivo xenograft tumor models

Twelve five‐week‐old female BALB/C nude mice were randomly assigned into two groups (*n* = 6). A packaged lentivirus with circ‐PKD2 was constructed by Genechem (Shanghai, China), while a scramble lentiviral vector was used as a control. A total of 1 × 10^7^ SCC-15 cells and CAL-27 cells were transfected with circ‐PKD2 or control lentivirus and injected subcutaneously into the posterior flank of nude mice. Two weeks after transfection, the mice were intraperitoneally injected with CDDP in PBS (10 mg/kg) once every week. Tumor volumes(V) were calculated from the length and width caliber measurement taken every four days using the formula: 0.5 × lengths × width^2^. After 4 weeks, the mice were sacrificed, and the tumors were collected, weighed, and photographed. Animal experiments were reviewed and approved by the Institutional Animal Care and Use Committee of the Affiliated Hospital of Qingdao University.

### Statistical analysis

All statistical analyses were performed using one-way analysis of variance (ANOVA), paired *t*-test, and independent *t*-test in the SPSS 22.0 software (IBM, Armonk, NY, USA). Data were expressed as mean ± SD. Statistical significance was defined as *p < 0.05, **p < 0.01.

## Results

### Cisplatin induces autophagy activation of OSCC cells

**Fig. 1:**
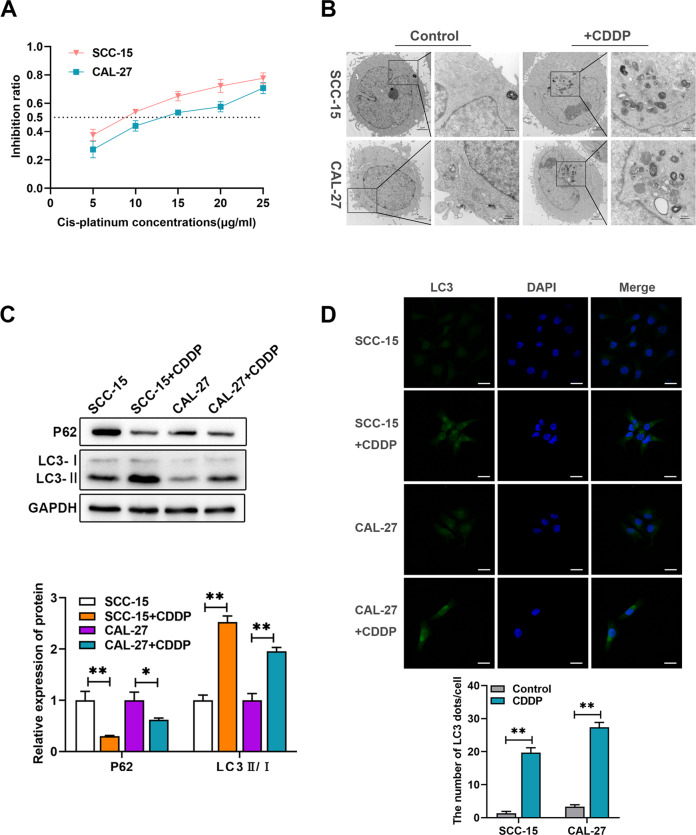
Cisplatin (CDDP) triggered autophagy in SCC-15 and CAL-27 cells. **A** The cisplatin inhibition ratio was determined through CCK-8 proliferation assays. **B** Micrographs of SCC-15 and CAL-27 cells treated with and without cisplatin for 24 h were taken using a transmission electron microscope. **C** Western blotting results of the expression levels of LC3B and P62 of SCC-15 and CAL-27 cells treated with cisplatin for 24 h. **D** Immunofluorescence results of LC3B distribution in SCC-15 and CAL-27 cells treated with cisplatin for 24 h. *p < 0.05, **p < 0.01.

Cisplatin chemotherapy induces autophagy in tumor cells, regulating tumor cells’ sensitivity and drug resistance to cisplatin [19]. To investigate the effect of cisplatin on OCSS cells, we evaluated the IC50 of SCC-15 and CAL-27 cells treated with cisplatin (Fig. 1A) followed by TEM and western blotting analysis to assess the changes in autophagosomes. Transmission electron microscopy revealed a substantial increase in accumulated autophagic vesicles in SCC-15 and CAL-27 cells treated with cisplatin at IC50 for 24 h compared to the untreated cells (Fig. 1B). In addition, the ratio of LC3-II to LC3-I in cisplatin-treated cells was approximately twice as high as that of untreated cells, while the expression of p62 decreased by about half (Fig. 1C). The level of LC3II was also significantly increased in the cisplatin-treated groups based on the immunofluorescence assay (Fig. 1D). Overall, the autophagy level of OSCC was significantly increased following cisplatin-treated, suggesting that autophagy regulates cisplatin sensitivity in OSCC cells.

### circ-PKD2 is key in cisplatin-induced autophagy activation

**Fig. 2:**
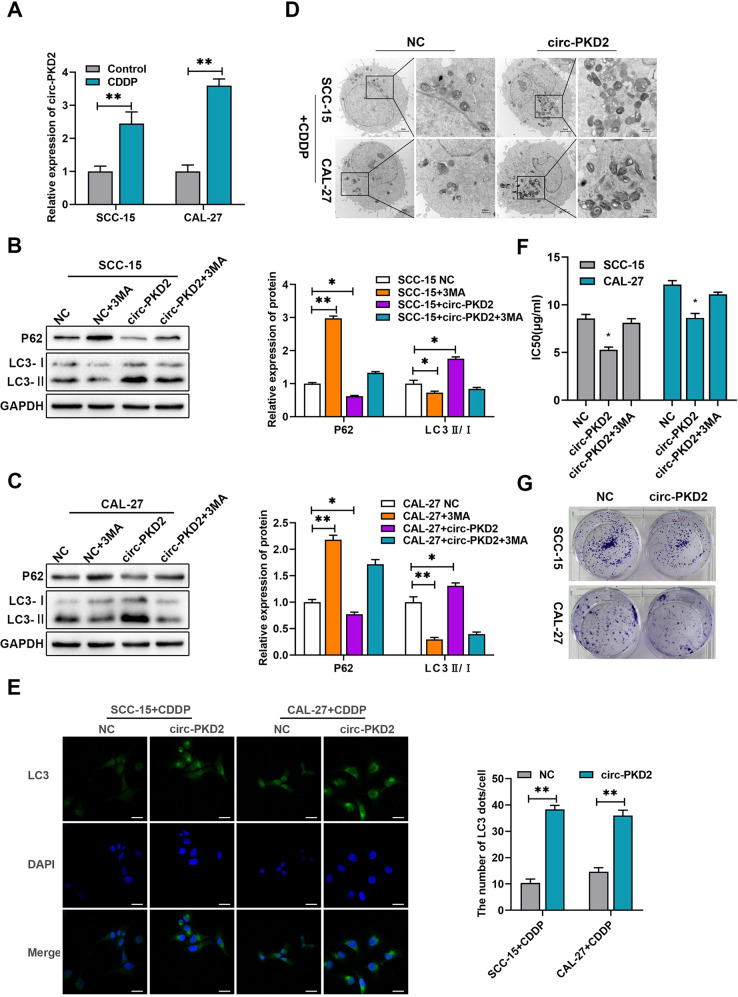
circ-PKD2 promoted autophagy in SCC-15 and CAL-27 cells and increased cisplatin sensitivity. **A** Relative mRNA levels of circ-PKD2 in SCC-15 and CAL-27 cells treated with and without cisplatin were detected using qRT‐PCR. **B**, **C** Western blotting results of LC3B and P62 expression in SCC-15 and CAL-27 cells treated with cisplatin and transfected with and without circ-PKD2 overexpression, and treatment with and without 3-MA(2 m mol/L) for 24 h. **D** Micrographs were taken using a transmission electron microscope showing autophagy in SCC-15 and CAL-27 transfected with circ-PKD2 overexpression and control-treated with cisplatin. **E** Immunofluorescence results revealing autophagy based on the distribution of LC3B in SCC-15 and CAL-27 cells transfected with and without circ-PKD2 overexpression and subsequently treated with cisplatin. **F** CCK-8 proliferation assay results show the IC50 of cisplatin in SCC-15 and CAL-27 cells with and without circ-PKD2 overexpression and 3-MA treatment. **G** The detected clonal formation rate after transfection with circ-PKD2 overexpression and control through cisplatin treatment. *p < 0.05, **p < 0.01.

Circ-PKD2 is significantly downregulated in OSCC cells, with overexpression of circ-PKD2 in vitro significantly inhibited the proliferation, migration, and invasion of OSCC cells [18]. To establish whether circ-PKD2 is involved in the regulation of OSCC cisplatin sensitivity, we investigated the effects of cisplatin on circ-PKD2 in SCC-15 and CAL-27 cells. The qRT-PCR results revealed that cisplatin caused a ~3 fold increase of circ-PKD2 in SCC-15 and CAL-27 cells compared to the untreated cells (Fig. 2A). In addition, the overexpression of circ-PKD2 greatly increased autophagy, evidenced by the increased LC3-II to LC3-I ratio and the attenuated p62 protein level (Fig. 2B, C). Based on TEM, the overexpression of circ-PKD2 promoted the accumulation of autophagic vesicles in SCC-15 and CAL-27 cisplatin-treated cells (Fig. 2D). The immunofluorescence assay confirmed that cells transfected with circ-PKD2 overexpression plasmid had a higher LC3II expression than the negative control treated with cisplatin (Fig. 2E). In addition, through CCK8 assay revealed that the overexpression of circ-PKD2 reduced IC50 of SCC-15 and CAL-27 cells but was reversed using the 3MA, a classic, widely used autophagy inhibitor (Fig. 2F). At the same time, treatment with low-dose cisplatin led to an increased clone formation rate in the over-expressed circ-PKD2 group (Fig. 2G). Thus, we conjectured that circ-PKD2 regulates the cisplatin sensitivity of OSCC cells through autophagy.

### miR-646 is a downstream effector in circ-PKD2-mediated autophagy in OSCC cells

**Fig. 3:**
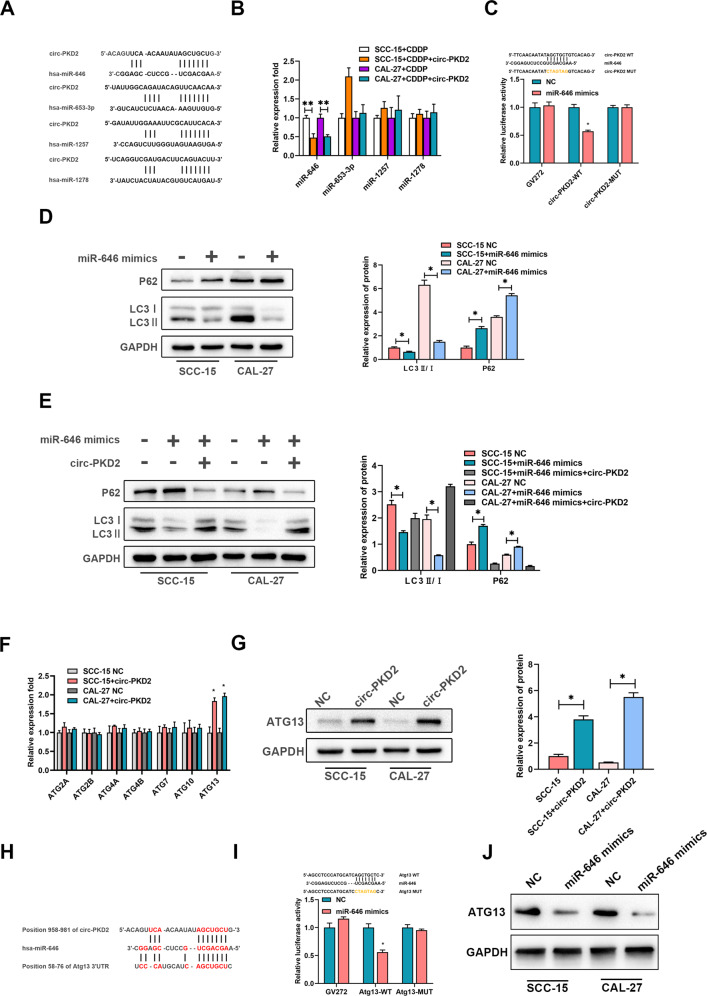
circ-PKD2 is a molecular sponge for miR-646. **A** A schematic graph of the four targets miRNAs-binding sequence. **B** The mRNA expression results of miRNAs measured using real-time PCR in SCC-15 and CAL-27 cells stably transfected with circ-PKD2 overexpression and controlled with cisplatin for 24 h. **C** The luciferase activity reporter assay results show the miR-646 binding site on circ-PKD2 predicted using a Circular RNA Interactome. **D** Western blotting results of cells transfected with miR-646 mimics and NC to reveal the autophagy-related proteins. **E** Western blotting results of cells co-transfected with miR-646 and circ-PKD2 to examine the protein levels. **F** The mRNA expression results of the autophagy-related genes were measured using real-time PCR in SCC-15 and CAL-27 cells transfected with circ-PKD2 overexpression and their control. **G** The protein levels of ATG13 in SCC-15 and CAL-27 cells transfected with circ-PKD2 and their control. **H** Illustration of the base pairing between miR-646 and circ-PKD2, and miR-646 and ATG13. **I** The reporter activity between miR-646 and Atg13 after normalization with respect to Renilla luciferase activity. *p < 0.05, **p < 0.01.

To identify the underlying mechanism of circ-PKD2-mediated autophagy, we performed bioinformatics analysis with TargetScan, CircInteractome, and starbase v2.0, which revealed that circ-PKD2 formed complementary base pairs with miR-646, miR-653-3p, miR-1257, and miR-1278 (Fig. 3A). The qRT-PCR results on the effect of circ-PKD2 on these miRNAs expression levels revealed that circ-PKD2 overexpression significantly decreased the miR-646 level in both SCC-15 and CAL-27 cells (Fig. 3B), while the miR-653-3p level was significantly inconsistent in the two cell lines. Meanwhile, there was no difference in the miR-1257 and miR-1278 expression levels. It has been reported that the circRNAs regulate chemotherapy resistance through the miRNA sponge adsorption [20]. To validate the relationship between circ-PKD2 and miR-646, a dual-luciferase reporter assay was performed, where miR-646 mimic was co-transfected with circ-PKD2 wild type (WT) or mutated (MUT) circ-PKD2 in the OSCC cells. The findings revealed that ectopic miR-646 expression significantly reduced the luciferase intensity of the circ-PKD2 wild type r but not the mutated circ-PKD2 in the OSCC cells (Fig. 3C). To explore the possible direct interaction between circ-PKD2 and miR-646, the role of miR-646 in autophagy in OSCC was evaluated where the SCC-15 and CAL-27 cells were transfected with miR-646 mimics. A declined LC3B (LC3II/LC3I) and risen p62 expression level was observed in cells transfected with miR-646 mimics compared to the negative control cells (Fig. 3D). Furthermore, decreased autophagy was observed in LC3B (LC3 II/LC3 I), while increased protein expression was observed in OSCC cells overexpressed with circ-PKD2 and miR-646 mimics (Fig. 3E). These findings confirmed that circ-PKD2 acted as a miR-646 sponge in OSCC cells. To establish the effect of circ-PKD2 on autophagy-related genes, the qRT-PCR results revealed that circ-PKD2 overexpression greatly enhanced the Atg13 mRNA level (Fig. 3F). Moreover, western blotting analysis revealed increased circ-PKD2 significantly up-regulated the ATG13 protein level (Fig. 3G). Bioinformatics analysis revealed that miR-646 was a good binding site to Atg13; thus, a WT and MUT luciferase reporter vectors of Atg13 were constructed (Fig. 3H), confirming a direct interaction between miR-646 and Atg13 (Fig. 3I). Furthermore, Western blotting revealed that miR-646 overexpression downregulated the Atg13 protein expression in SCC-15 and CAL-27 cells (Fig. 3J). Overall, circ-PKD2 inhibited the inhibitory effect of miR-646 on Atg13, promoting autophagy.

### circ-PKD2 promotes the cisplatin sensitivity of OSCC cells through Atg13

**Fig. 4:**
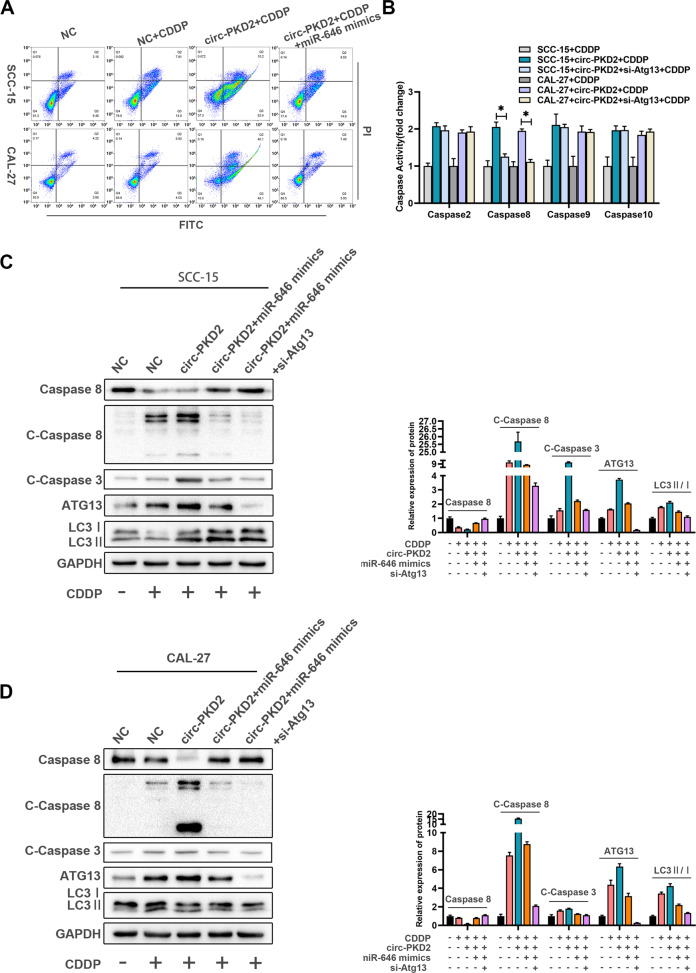
circ-PKD2 activated caspase-8 through Atg13 to increase the sensitivity of oral squamous carcinoma cells to cisplatin. **A** Flow cytometry results showing the apoptosis rate of cells transfected with circ-PKD2 overexpression, circ-PKD2 + miR-646 mimics, and circ-PKD2 + miR-646 mimics+si-Atg13. **B** Real-time PCR results showing the caspases’ activities. **C** Western blotting results showing the activation of caspase-8, caspase-3, ATG13, P62, and LC3B in SCC-15 cells transfected with circ-PKD2 overexpression, circ-PKD2 + miR-646 mimics, and circ-PKD2 + miR-646 mimics+si-Atg13. **D** Western blotting results showing the activation of caspase-8, caspase-3, ATG13, P62, and LC3B in CAL-27 cells transfected with circ-PKD2 overexpression, circ-PKD2 + miR-646 mimics, and circ-PKD2 + miR-646 mimics+si-Atg13. *p < 0.05, **p < 0.01.

To confirm the relationship among circ-PKD2, miR-646, and Atg13 in the cisplatin sensitivity, miR-646 was overexpressed, which led to increased apoptosis. However, the overexpression of circ-PKD2 was attenuated in the presence of miR-646 mimics (Fig. 4A). This implies that the increased cisplatin sensitivity caused by circ-PKD2 was due to the increased miR-646. Given that the target relationship between miR-646 and Atg13 has been identified, we hypothesized that circ-PKD2 ultimately regulates cisplatin sensitivity by up-regulating Atg13. Hsin et al. found that GMI improved apoptosis *via* autophagy, which regulated chemotherapy sensitivity in lung cancer cells [21]. Besides, with the complex regulatory relationship between autophagy and apoptosis, we wanted to explore whether changes in cisplatin sensitivity induced by circ-PKD2 resulted from the regulation between autophagy and apoptosis. Moreover, caspase-2, 8, 9, and 10 are initiators, and caspase-3 is the most important executor of apoptosis. To explore whether circ-PKD2-induced cisplatin sensitivity was due to the activation of caspase members, qRT-PCR was used to detect the caspase’s mRNA levels. The results showed that Atg13 knockdown attenuated the activity of caspase-8 induced by overexpression of circ-PKD2 (Fig. 4B). In addition, the Western blotting revealed that circ-PKD2 significantly increased the cleavage of caspase-8 and caspase-3 caused by cisplatin but was attenuated by miR-646 mimics and si-Atg13 (Fig. 4C, D). Overall, circ-PKD2 upregulated ATG13 by sponging miR-646 and activated caspase-8 and caspase-3, promoting apoptosis and increasing cisplatin sensitivity under the effect of cisplatin in OSCC cells.

### Overexpression of circ‐PKD2 upregulates the cisplatin sensitivity of OSCC cells in vivo

**Fig. 5:**
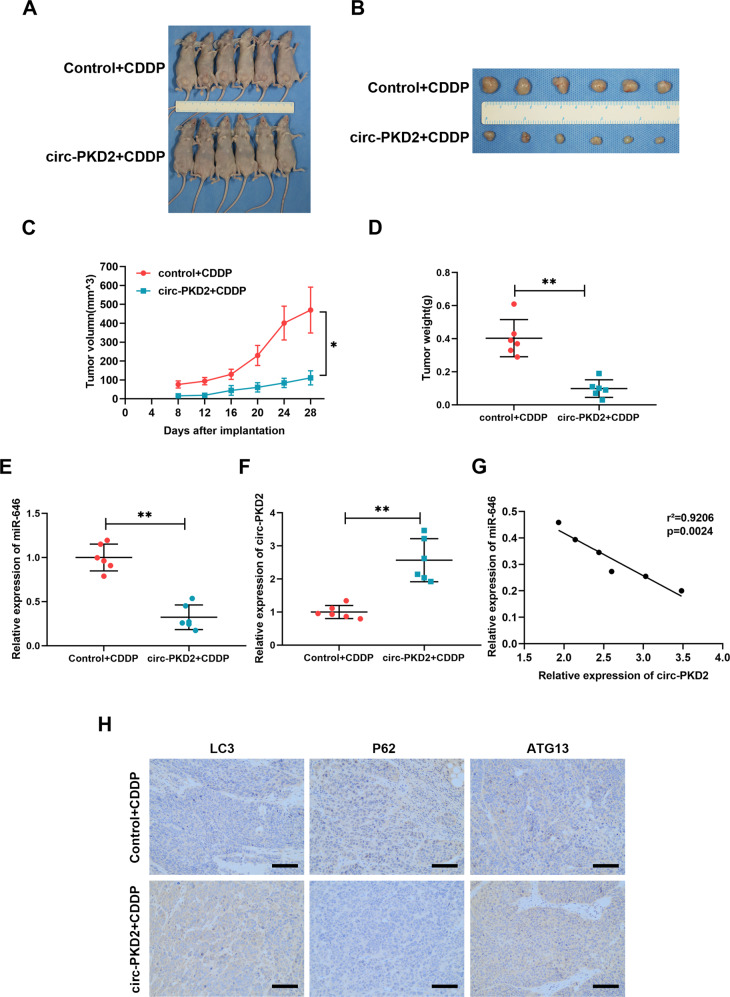
Effect of circ-PKD2 overexpression on the sensitivity of cisplatin. **A**–**D** Tumor volumes, weight, and tumor growth curves of subcutaneous implantation models of SCC-15 cells. **E** The expression of miR-646. **F** The expression of circ-PKD2. **G** qRT‐PCR results are showing the correlation between circ-PKD2 and miR-646. **H** IHC results show the expression of LC3B, P62, and ATG13 in the circ-PKD2 overexpressing and control groups. *p < 0.05, **p < 0.01.

The xenograft model was used to confirm the effect of circ-PKD2 on the chemosensitivity of OSCC cells in vivo. Consistent with in vitro observations, circ-PKD2 overexpression significantly increased the chemosensitivity evident as reduced tumor volume and weight (Fig. 5A–D). Based on the qRT‐PCR data, circ-PKD2 in the circ-PKD2 group was increased while miR‐646 was decreased compared with the control group (Fig. 5E, F). At the same time, there was a negative correlation between the two expressions (Fig. 5G). Furthermore, immunohistochemistry analysis revealed that the tumors in LC3B and ATG13 were increased by overexpression of circ-PKD2 compared with the control while the expression of p62 was decreased (Fig. 5H). This confirmes the essential roles of circ-PKD2 in regulating autophagy which is partially the underlying mechanism of circ-PKD2-mediated sensitivity of cisplatin in OSCC cells.

## Discussions

**Fig. 6:**
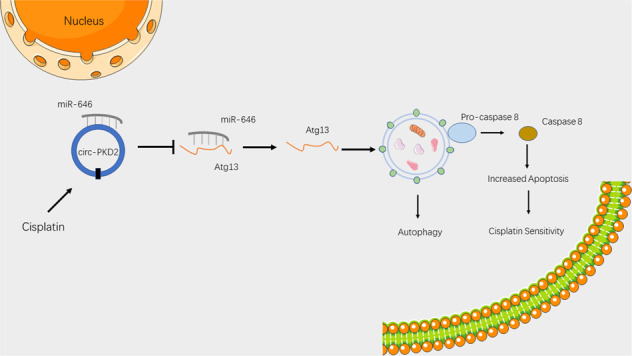
Circ-PKD2 promotes Atg13-mediated autophagy by inhibiting miR-646 to increase the sensitivity of cisplatin. Schematic diagram depicting the proposed model in which circ-PKD2 regulates autophagy and cisplatin sensitivity.

Cisplatin is the most commonly used chemotherapeutic medication in treating many types of malignancies, including head and neck solid tumors [22]. However, decreasing sensitivity is an obvious challenge; thus, it is particularly important to explore a strategy to improve chemotherapy sensitivity. Although current research has focused on the molecular mechanisms of chemosensitivity [23], the underlying mechanisms remain largely unknown. However, recent studies have established that autophagy plays an essential role in the regulation of chemotherapy sensitivity [24, 25]. In addition, circRNA regulates the sensitivity of cisplatin in lung cancer [26]. The present study established that circ-PKD2 promotes the Atg13 expression by sponging miR-646 to accelerate cisplatin sensitivity (Fig. 6).

CircRNA is a non-coding RNA discovered in recent years which plays an important role in regulating human tumors development [27, 28]. At the same time, the association between chemotherapy sensitivity and abnormal circRNA expression in various malignant tumors has been revealed [29–31]. However, the mechanism of cisplatin sensitivity in OSCC is still unclear. Nevertheless, circ-PKD2 expression is down-regulated in OSCC cells based on the microarray tool, where its overexpression regulated the proliferation, migration, and invasion of OSCC cells [18]. Similarly, in the present study, circ-PKD2, as a tumor suppressor gene promoted the sensitivity of OSCC cells to cisplatin.

CircRNAs regulate protein translation processes by acting as miRNA sponges, regulating RNA–protein interactions and RNA splicing [32]. For example, circEYA1 RNAs function as efficient miR-582-3p sponges suppressing cervical adenocarcinoma [33]. In addition, circ-PRMT5 acts as a miR-509-3p sponge to promote breast cancer [34]. In addition, circRNAs regulate miRNAs through the complementary binding sites [35, 36] and chemotherapeutic resistance and sensitivity of tumor cells through miRNA [30, 37, 38]. This study used bioinformatics and dual-luciferase reporting assay to investigate whether circ-PKD2 had a good base complementarity relationship with miR-646. The findings suggested that circ-PKD2 was involved in regulating chemotherapy sensitivity in OSCC cells mainly through the miR-646 adsorption.

Autophagy is a conservative cellular metabolic process by the lysosomes degradation of cellular components to maintain normal physiological activities and homeostasis. In healthy cells, autophagy maintains homeostasis and clears away damaged organelles within the cells. However, when induced by environmental pressures such as nutrient deficiency or hypoxia, autophagy degrades non-essential or damaged proteins and organelles to reuse the resulting amino acids in more important cellular processes [39]. In cancer treatment, this adaptation is a double-edged sword [40]. Autophagy desensitizes cells to stress conditions such as chemotherapy or nutrition, evidenced in many tumor microenvironments [41], with prolonged autophagy leading to autophagic cell death [42]. In mammals, autophagy can either be microautophagy, chaperon-mediated autophagy, or macrophages [43, 44], based on how the cell degrades the substrate into the lysosome. The autophagy involved in this study is the macrophage. Many autophagy-related proteins are involved in autophagy, with Atg1/ULK complex being one of the most upstream factors in the formation of autophagosomes [45, 46].

Atg13 is an indispensable component of the Atg1/ULK complex. With autophagy and apoptosis sharing many upstream pathways which regulate each other [47], we speculated circ-PKD2 played a role in increasing the sensitivity of cisplatin following the downstream apoptosis induced by autophagy, rather than the production of protective autophagy in OSCC cells. In the present study, circ-PKD2 increases the Atg13 expression, activating caspase-8 to aggravate apoptosis in OSCC cells. This is consistent with Ding et al. [48], who established that Atg13 interacts with FADD to activate caspase-8, where histamine regulates apoptosis under hypoxia. Although we have confirmed the regulatory relationship between circ-PKD2 and cisplatin sensitivity in OSCC cells, the association between circ-PKD2 expression and clinical chemotherapeutic characteristics of OSCC patients should be confirmed with a large cohort.

In conclusion, our study established the role of circ-PKD2 in the chemosensitivity of OSCC cells. Circ-PKD2 promotes autophagy by sequestering miR-646 and tittering miR-646 off its target Atg13, thus increasing the Atg13 level and activating caspase-8 to aggravate apoptosis in OSCC cells. These findings provide novel insights into the molecular mechanisms underlying chemosensitivity.

## Supplementary information

Reproducibility checklist

List of primers

## Data Availability

The datasets generated and/or analyzed during the current study are available from the corresponding author on reasonable request.
