# Supplementary material for: Circ-PKD2 promotes Atg13-mediated autophagy by inhibiting miR-646 to increase the sensitivity of cisplatin in oral squamous cell carcinomas
Source: Cell Death Dis. 2022 Feb 26;13(2):192. doi: 10.1038/s41419-021-04497-8 (PMC8882170; doi:10.1038/s41419-021-04497-8)
Supplement: Supplementary file 2 — List of primers [file 41419_2021_4497_MOESM2_ESM.docx]

Table S1. List of primers.

| Gene | Sequence |
| --- | --- |
| Hsa_circ_0070401 | F: CATTATTTTCCTAGCGTATGCTCAGT  R: CTGTGACATCATCCGGGTGTAG |
| Hsa_miR_1257 | F: GGGGAGTGAATGATGGGTTCT  R:GTCGTATCCAGTGCAGGGTCCGAGGTATTCGCACTGGATACGACGGTCAG |
| Hsa_miR_1278 | F: GGGCCCTAGTACTGTGCATATCAT  R:GTCGTATCCAGTGCAGGGTCCGAGGTATTCGCACTGGATACGACATAGAT |
| Hsa_miR_646 | F: GGGAAGCAGCTGCCTCTG  R:GTCGTATCCAGTGCAGGGTCCGAGGTATTCGCACTGGATACGACGCCTCA |
| Hsa_miR_653-3p | F: GGGGTTCACTGGAGTTTGTTTC  R:GTCGTATCCAGTGCAGGGTCCGAGGTATTCGCACTGGATACGACTATTGA |
| ATG2A | F: TGGCAGCTTAGGGTCATGCT  R: AGTAACCGTCCCTCACAACGA |
| ATG2B | F: ATCGAGCCCAAGAAGACCAA  R: TTCTCGAGCCGCAGTTTCAT |
| ATG4A | F: GCATTGGGATTTTTCTGCAAA  R: TGGCTTGGCTGGAGGTACA |
| ATG4B | F: TGGCGAAGGCAAGTCCATAG  R: CACAACCTTCTGATTTCCTCCAT |
| ATG7 | F: AGCTCGCTTAACATTGGAGTTCA  R: TTTGCTGCTTGTTCCAAAAGG |
| ATG10 | F: TCATCCCTGCAAGACGAATG  R: GACGTTGCTTTGGCATAACTCA |
| ATG13 | F: TAAAGATGACATTCTTCCGATGGA  R: TTCTCATGCACAGCCAGCTT |
| Caspase2 | F: TTCTGAGCGGGCTTGTGATA  R: TAGAGGTGGCGGCACAGAGT |
| Caspase8 | F: TGAGGTCTTTTAAGTTTCTTTTGCAA  R: TTTGGGCACAGACTCTTTTCAG |
| Caspase9 | F: GCCAGGCAGCTGATCATAGATC  R: TTCTAGGGTTGGCTTCGACAA |
| Caspase10 | F: TGCAGCACCTCAACTGTACCA  R: GCGAGTCTTTCAGAAGGAAGATCA |
| U6 | F: ACGAATACCGGCGTGAGAAA  R: TCGTGAAAGACCGCAGCAAA |
| GAPDH | F: TCAAGGCTGAGAACGGGAAG  R: TCGCCCCACTTGATTTTGGA |
